# Ethanol-Induced Autophagy in Sertoli Cells Is Specifically Marked at Androgen-Dependent Stages of the Spermatogenic Cycle: Potential Mechanisms and Implications

**DOI:** 10.3390/ijms20010184

**Published:** 2019-01-06

**Authors:** Akio Horibe, Nabil Eid, Yuko Ito, Yoshinori Otsuki, Yoichi Kondo

**Affiliations:** 1 Kubomizuki Lady’s Clinic 3-13-8, Mikatadai, Nishi-ku, Kobe, Hyogo 651-2277, Japan; horibe@kubomizuki.or.jp; 2 Department of Anatomy and Cell Biology, Division of Life Sciences, Osaka Medical College, 2-7 Daigaku-machi, Takatsuki, Osaka 569-8686, Japan; an1006@osaka-med.ac.jp (Y.I.); konchan@osaka-med.ac.jp (Y.K.); 3 Osaka Medical College, Takatsuki, Osaka 569-8686, Japan; y.otsuki@osaka-med.ac.jp

**Keywords:** ethanol, autophagy, LC3, apoptosis, Sertoli, androgen receptor, PINK1, sperm retention, spermatogenic cycle, mitophagy, lipophagy, LAP, infertility

## Abstract

In a recent study, we reported that acute ethanol exposure enhanced autophagy in Sertoli cells (SCs) of adult rats. However, further research is needed to clarify the specific spermatogenic stage exhibiting the highest autophagic response, the mechanisms behind such specificity, and the related relevance to sperm. This brief report provides results indicating that stages VII–VIII (androgen-dependent or spermiation stages) of the spermatogenic cycle exhibited more marked autophagic response in acute-ethanol treated rats (ETRs) than other stages based on suppression of androgen receptor (AR), analysis of microtubule-associated protein 1 light chain 3 (LC3) (an autophagosomal marker) immunostaining in SCs, double labeling of LC3 and lysosomal proteins and electron microscopy. Ultrastructural observations and TUNEL method revealed a notable presence of phagocytosed apoptotic germ cells and retained sperm in SCs of ETRs at these specific stages—a finding rarely observed in control testes. In addition, PTEN-induced putative kinase 1 ( PINK1) (a sensor of mitochondrial damage and mitophagy) and giant lipid droplets were found to have accumulated in SCs of ETRs at same stages. Our data show novel findings indicating that stages VII–VIII of the spermatogenic cycle exhibit high levels of autophagy, specifically under stress conditions, as expressed by the term *autophagic stages*. This stage-specific upregulation of autophagy in SCs may be related to AR suppression, mitochondrial damage, lipid accumulation, and phagocytosis of apoptotic cells. The phenomenon may be an essential part of ensuring the viability of SCs and supporting germ cells in toxic environments.

## 1. Introduction

Autophagy is a survival pathway for the clearance and recycling of damaged cellular components within autophagic vacuoles (AVs) upon exposure to various stressors such as hypoxia, oxidative stress, mitochondrial damage, and lipid droplet (LD) accumulation. This recycling of autophagic cargo provides sources of energy or building blocks for the synthesis of macromolecules [1,2]. Morphologically, autophagy is characterized by the formation of isolation membranes that engulf a region of the cell forming early AVs or autophagosomes, which are mediated by LC3 (a specific autophagosome marker). The latter then fuse with lysosomes to form late AVs or autolysosomes, and the contents are degraded by cathepsins [3,4]. Autophagy may be a selective process in the clearance of damaged mitochondria (mitophagy) or excessive lipid droplets (lipophagy) in hepatocytes in association with exposure to acute or chronic ethanol intake. Under such exposure conditions, PINK1 accumulation in mitochondria is widely accepted as a sensor of mitochondrial damage and an activator of anti-apoptotic mitophagy via a Parkin-related pathway [2,3,5,6].

Various roles played by autophagy have been recently reported in relation to Sertoli cells (SCs). First, the process is required for efficient clearance of apoptotic germ cells by SCs in mice based on LC3-associated phagocytosis (LAP) [7]. Second, autophagy degrades androgen-binding protein (ABP) in rat SCs under the control of testosterone (a negative regulator in autophagic degradation of ABP) [8]. Third, autophagy is an essential element of ectoplasmic specialization (ES) assembly in mouse SCs (important for sperm release and residual body (RB) formation in the spermiation stage). Disruption of autophagy (via knocking down autophagy genes) in SCs has been found to result in enhanced germ cell apoptosis, reduced spermatozoa counts and abnormal spermiation [9]. Fourth, germ cells and spermatozoa, in particular, depend on lactate production by SCs via autophagy and glycolysis for viability and motility under normal and stressed conditions [10,11]. Fifth, autophagy upregulation has been found to be a prosurvival mechanism for cultured SCs upon exposure to heat stress [11] or high dose of ethanol [4].

Excessive alcohol intake has been reported to enhance germ cell apoptosis, resulting in infertility problems via mechanisms related to oxidative stress, upregulation of inducible nitric oxide synthase (iNOS) and mitochondrial dysfunction [4,5,6]. The authors recently reported that acute ethanol exposure upregulated autophagy in SCs of adult rats, and that this was accompanied by elevated germ cell apoptosis, androgen receptor (AR) suppression in testicular somatic cells and enhanced expression of iNOS in SCs [4]. The rat spermatogenic cycle includes 14 stages, each involving specific morphological and functional criteria (simplified into four groups consisting of stages I–VI, VII–VIII, IX–XI and XII–XIV) [12]. However, the above-mentioned literature [7,8,9,10,11] on autophagy in SCs, as well as a recent report by the current authors [4], does not provide a detailed analysis of stage-specific autophagic response in SCs under normal conditions or stress-related situations such as those associated with ethanol exposure. Further clarification is also needed regarding the potential mechanisms behind the stage-specificity of autophagy in SCs and related implications for germ cells and fertility. In this paper, the authors provide a brief report of investigation regarding the autophagic response of SCs to acute ethanol exposure in various stages of the spermatogenic cycle, the trigger mechanisms for such specificity, and associated clinical relevance.

## 2. Results

### 2.1. AR Expression Was at Its Highest in SCs during Stages VII–VIII in Control Testes, but Was Dramatically Suppressed in Association with Acute Ethanol Exposure

**Figure 1:**
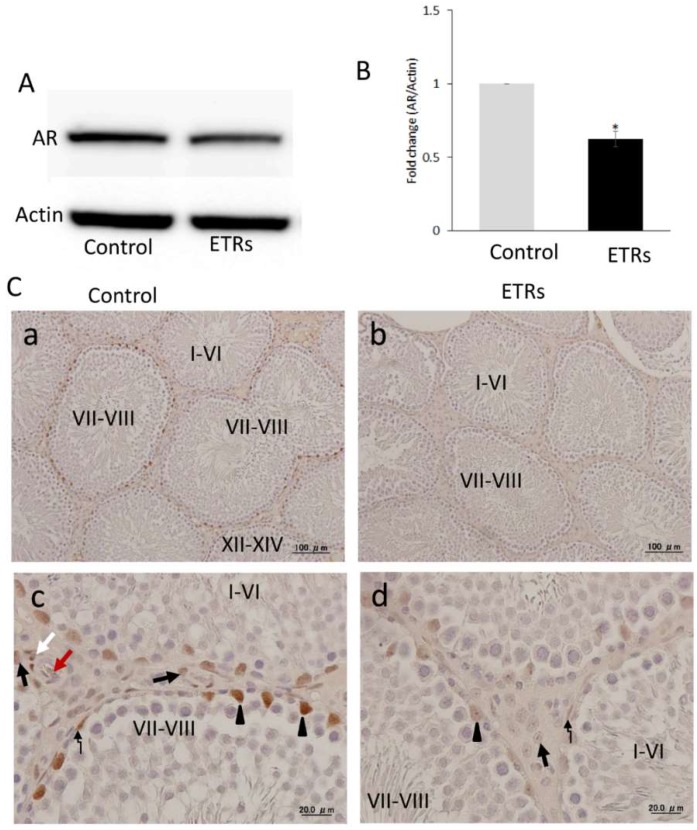
Ethanol-induced reduction of androgen receptor (AR) expression in ETRs testes. (**A**,**B**) Western blot analysis for AR. The relative expression level for protein was normalized to actin and expressed as fold change relative to the control (*n* = 3). * *p* < 0.01 compared to the control group. (**C**) Immunohistochemistry (IHC) of AR in control (a,c) and ETRs (b,d). Arrow heads indicate nuclear staining of AR in SCs, while broken arrows mark its expression in myoid cells. The black and white arrows indicate the expression of AR in Leydig cells and smooth muscles of blood vessels, respectively (red arrow: blood vessel lumen).

Western blot analysis of testicular lysates 24 h after a single intraperitoneal injection of ethanol (5 g/kg) in adult rats showed a statistically significant reduction of AR in ethanol-treated rats (ETRs) compared to the control group (Figure 1A,B). Immunohistochemistry (IHC) demonstrated that nuclear AR expression in control testes was detected in SCs and myoid cells in most stages, but was at its highest in stages VIII–VIII (Figure 1C(a,c)). AR expression was also observed in Leydig cells and smooth muscle of testicular blood vessels, as reported previously [13,14]. Meanwhile, a marked decrease in AR expression was evident in all somatic cells of ETR testes, including in all stages of the spermatogenic cycle (Figure 1C(b,d)). As VIII–VIII are known as spermiation- or androgen-dependent stages [15,16], the suppression of AR in ETR testes observed in the current study may be related to androgen suppression [14,17]. Because SC autophagy [9] and AR signaling via related expression by SCs and myoid cells [18] are essential for spermiation, germ cell survival and fertility under physiological conditions, the authors monitored stage-specific autophagic responses in SCs of ETRs and control testes.

### 2.2. Autophagy Is Specifically Upregulated in SCs of ETRs during Stages VII–VIII of the Spermatogenic Cycle

**Figure 2:**
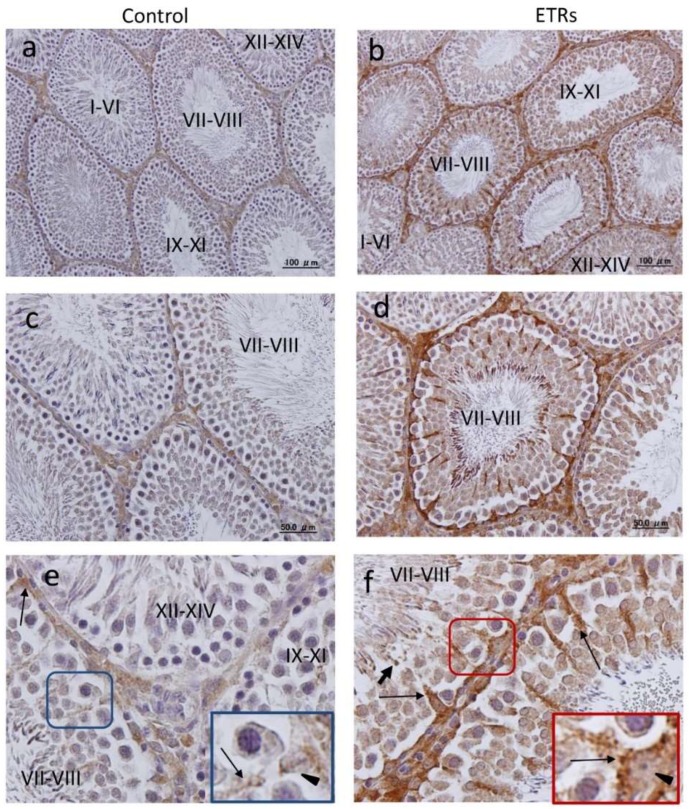
Immunolabeling for LC3 with highest expression in SCs of ETRs at stages VII–VIII of spermatogenic cycle. ((**a**,**c**,**e**) control group; (**b**,**d**,**f**) ETRs). Higher magnifications of framed areas in (**e**,**f**) showing LC3 puncta (long arrows) in SCs are shown in the insets. The short arrows indicate LC3 dots in RBs. Black arrow heads mark SC nuclei.

**Figure 3:**
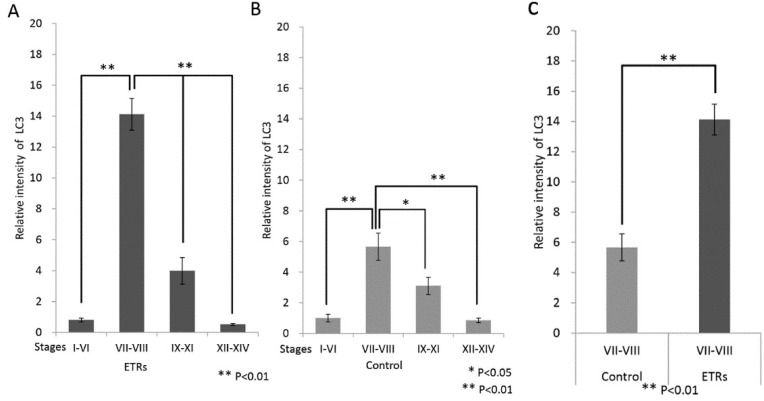
Quantification of LC3 expression in SCs at various stages of the spermatogenic cycle. (**A**) Histogram demonstrating quantification of LC3 intensity in SCs of ETRs at various stages based on IHC analysis from Image J software (highest in stages VII–VIII for ETRs); (**B**) Histogram showing LC3 quantification in control group; (**C**) Histogram demonstrating higher intensity of LC3 expression in stages VII–VIII in ETRs compared to the control. (* *p* < 0.05; ** *p* < 0.01) (*t*-test).

**Figure 4:**
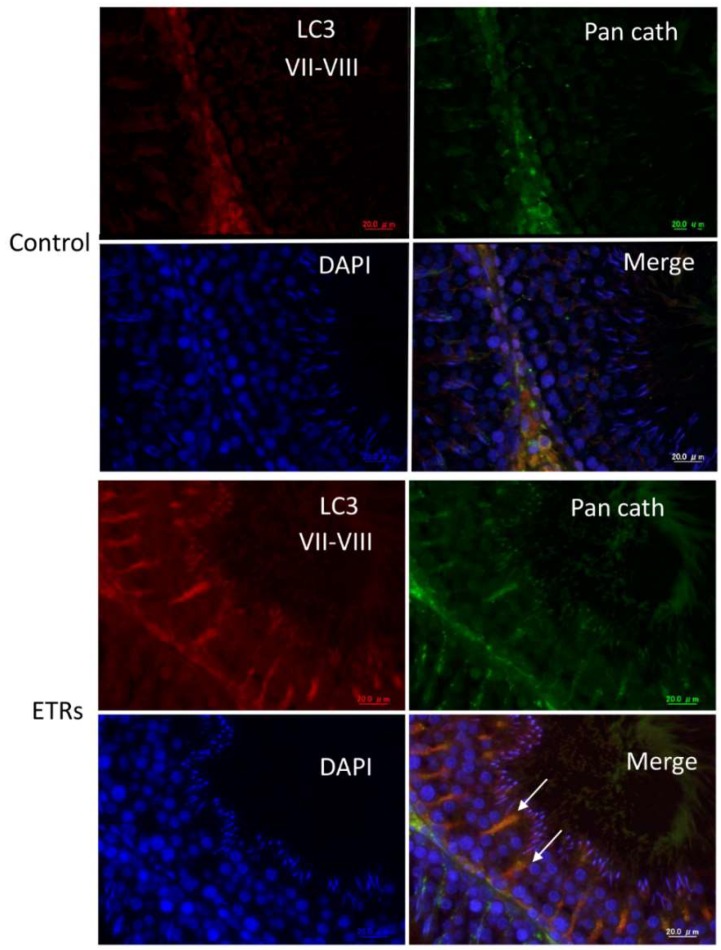
Immunofluorescence double-labeling for LC3 and pan cathepsin in stages VII–VIII of the spermatogenic cycle. Note the enhanced colocalization of LC3 (red) and pan cathepsin (green) in SCs of ETRs (white arrows). DAPI was used for nuclear counterstaining. Pan cath, pan cathepsin

**Figure 5:**
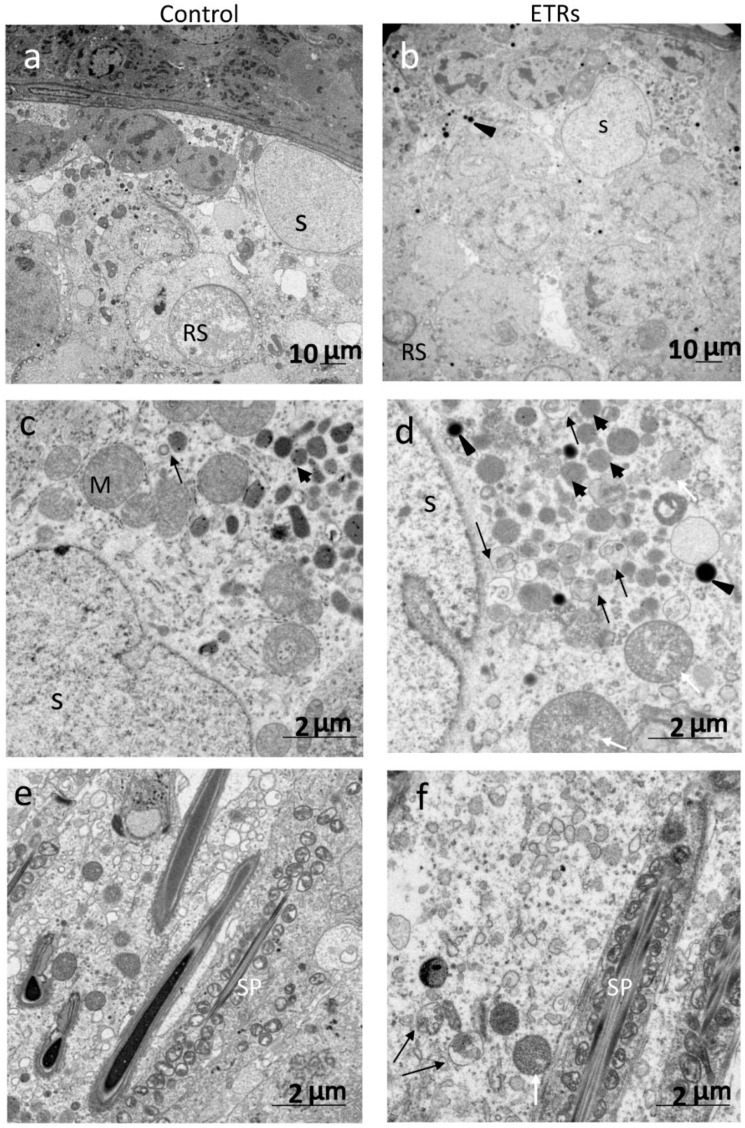
Ultrastructural features of enhanced autophagy in SCs of ETRs during stages VII–VIII. ((**a**,**c**,**e**): control testes; (**b**,**d**,**f**): ETRs). The long black arrows indicate autophagic vacuoles (AVs) containing membranous structures, while the white arrows mark damaged mitochondria. The short black arrows and arrow heads show lysosomes and lipid droplets (LDs), respectively. RS: round spermatid; S: SC nucleus; SP: sperm; M: mitochondrion.

The IHC information in Figure 2 clearly demonstrates that SCs of ETRs at stages VII–VII exhibited the highest expression of LC3 (e,f). Higher magnification also clearly shows enhanced LC3 puncta formation in SCs and RBs of ETRs at the same stages compared to very low levels in the control group, indicating enhanced autophagosome formation (mediated by LC3-II) as previously reported by the authors [2,3,4]. In addition, enhanced LC3 expression was noted in testicular interstitial cells of ETRs. This IHC of LC3 was confirmed by Western blot analysis (data not shown). Quantitative analysis of LC3 expression in SCs of ETRs (Figure 3A) confirmed IHC with significantly higher LC3 intensity in stages VII–VIII than in other stages. This trend of autophagy-related stage-specificity was also seen in SCs of control testes (Figure 3B) (stages VII-VIII showed the highest LC3 intensity). Figure 3C shows statistically higher LC3 intensity in ETR SCs in stages VII–VIII than in the control group. Immunofluorescence (IF) double labeling of LC3 with pan cathepsin (a lysosomal marker) (Figure 4) showed enhanced colocalization in SCs of ETRs in stages VII–VIII, indicating enhanced autolysome formation and flux activity [2,3,4]. This enhanced flux activity was supported by colocalization of LC3 and p62 (data not shown). Transmission electron microscopy (TEM) (Figure 5) confirmed the above-mentioned IHC and IF findings related to stage-specific upregulation of autophagy. As shown in Figure 5, and compared to the control group (a,c,e), greater accumulation of AVs (autophagosomes and autolysosomes) was seen in SCs of ETRs (b,d,f). These AVs were located in the perinuclear (d) and apical regions (f) close to the sperm midpiece, and were associated with damaged mitochondria and numerous LDs, which were occasionally very large as seen in the figure below.

### 2.3. Enhanced Autophagy in SCs of ETRs during Stages VII–VIII Is Associated with Germ Cell Apoptosis, Sperm Retention and Accumulation of PINK1 and Large LDs

**Figure 6:**
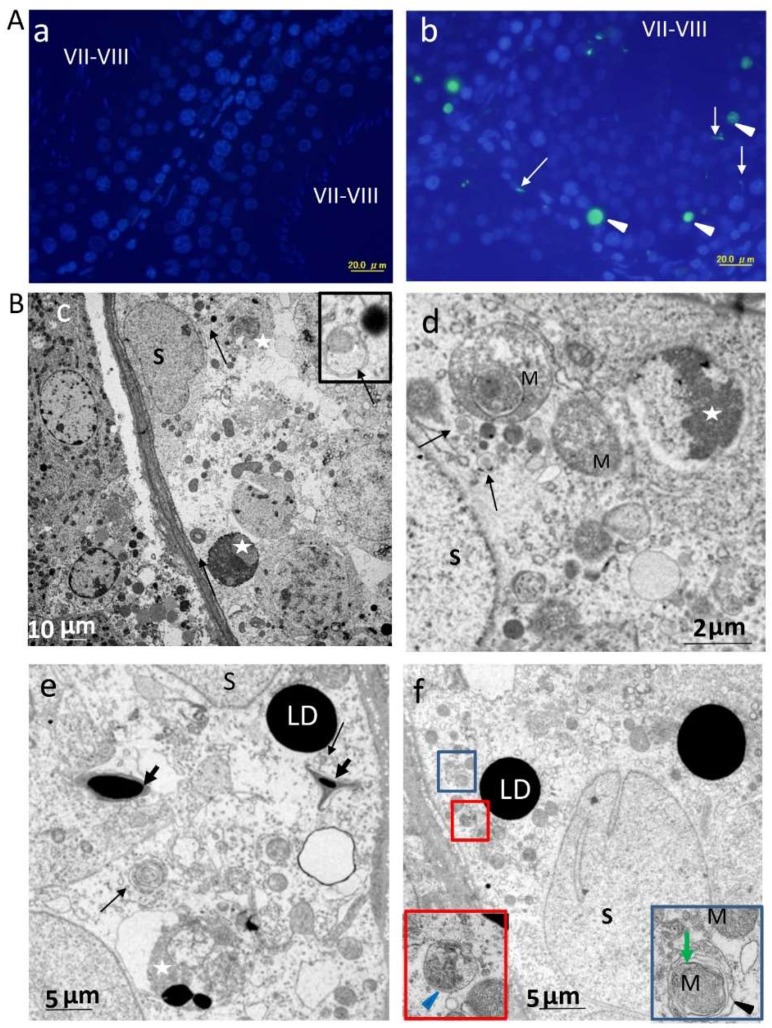
Accumulation of apoptotic germ cells, retained sperm, damaged mitochondria and large LDs in SCs of ETRs at stages VII-VIII. (**A**) TUNEL labeling of apoptotic cells (white arrow heads) in control (a) and ETR (b) testes. The white arrows mark retained sperm with TUNEL positivity apparently phagocytosed by SCs. (**B**) TEM showing apoptotic germ cells (stars in c–e) and retained sperm (short arrows in e) phagocytosed by SCs of ETRs. The insets in f are higher magnifications of the boxed areas, demonstrating a mitophagosome marked by green arrow (right inset) and autolysosome marked by blue arrow head (left inset). The long black arrows indicate AVs, while the black arrow head in the right inset marks autophagosomal membrane. M: damaged mitochondria; S: SC nucleus; LD: lipid droplet.

**Figure 7:**
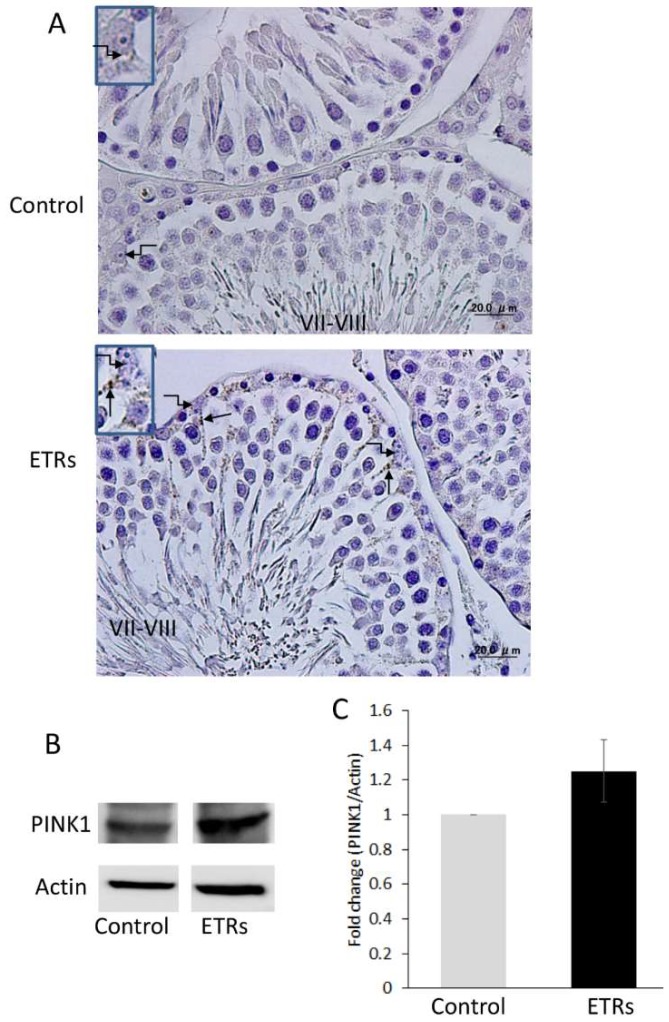
Overexpression of PINK1 in SCs of ETRs indicating mitochondrial damage. (**A**) IHC of PINK1. Note the perinuclear localization of PINK1 (arrows). The broken arrows indicate SC nuclei. The inset shows magnified SC nuclei; (**B**,**C**) Western blot analysis of PINK1. The relative expression level for protein was normalized to actin and expressed as fold change relative to the control (*n* = 3).

As autophagy in SCs was reportedly triggered by enhanced germ cell apoptosis via LAP [7], the authors analyzed apoptotic events in stages VII–VIII (Figure 6A,B). As shown in 6A (a), apoptotic germ cells were rarely detected at this stage, as reported in previous studies [15,19,20]. However, notable apoptosis of germ cells was observed in stages VII–VIII with the TUNEL method (Figure 6A(b)) and TEM (Figure 6B). Interestingly, some sperm (based on nuclear morphology) at the lumen of seminiferous tubules or retained within SCs showed TUNEL positivity. TEM revealed that apoptotic germ cells and retained spermatozoa were phagocytosed by SCs of ETRs, which may be related to AR-related spermiation failure [18]. This sperm retention was also detected in stages IX–XII (data not shown). Importantly, SCs of ETRs contained numerous AVs (shown by long black arrows in Figure 6c–e) adjacent to phagocytosed apoptotic germ cells and retained sperms, suggesting the potential of LAP [7]. LAP may be also required for clearance of phagocytosed cells and retained sperms at other stages. Damaged mitochondria exhibiting loss of cristae, irregularity of the outer mitochondrial membrane and dark deposits in the matrix were frequently seen in SCs of ETRs (Figure 6B(d)). One of these mitochondria was engulfed by an autophagosomal membrane resulting in mitophagosome formation (Figure 6B(f), inset), indicating the induction of mitophagy [6]. In addition, while LDs were not commonly seen in SCs of control testes at this specific stage [21], accumulation of numerous large LDs was observed in SCs of ETRs (Figure 6B(e,f)) (seen also in Figure 5), which may be related to enhanced phagocytosis of germ cells [21], androgen suppression [22,23] and associated mitochondrial damage [24]. This accumulation of LDs in testes of ETRs was immunohistochemically confirmed by specific markers (data not shown). The mitochondrial damage and mitophagy in SCs of ETRs were confirmed by immunohistochemical observation of a punctate pattern of perinuclear PINK1 accumulation, suggestive of mitochondrial localization [3], as shown in Figure 7A and supported by Western blot analysis of the whole testicular lysate (Figure 7B).

## 3. Discussion

This report details several novel findings. First, SCs of ETRs in stages VII–VIII exhibit high levels of autophagy, as expressed by the term *autophagic stages*. Second, this enhanced autophagic response in SCs relating to ethanol may be associated with AR suppression, mitochondrial damage, LD accumulation and enhanced phagocytosis of germ cells and retained sperm. Third, PINK1 accumulation in these SCs and the activation of mitophagy could be related to the Parkin signaling pathway. To the authors’ knowledge, this is the first report to show such findings in specific stages of spermatogenic cycle.

The specific ultrastructural features of SCs in stages VII–VIII in normal adult rat testes have been reported in detail previously [25], with observation that SCs in these stages had the highest number of mitochondria, the greatest presence of mitochondrial-ER contact sites, and peak levels of lysosomal density. This may correspond to the enhanced autophagy in SCs of ETRs observed in the same stages in the current study, as it has been reported that mitochondrial-ER contact sites are locations for autophagosome formation [26]. Increased lysosomal biogenesis via transcription factor EB (TFEB) nuclear translocation is also essential for enhancement of autophagy as recently reported by the authors [4], with high levels of nuclear TFEB expression in SCs of ETRs in stages VII–VIII and even higher levels in stage VI (data not shown).

Another important specific characteristic of this stage range concerns AR. Stages VII–VII, or the spermiation stages, are known to be AR-dependent, because they exhibit the highest levels of AR expression in SCs under normal conditions and are more sensitive to androgen suppression by Leydig cell toxicants (such as EDS) and genetic or hormonal manipulation [15,16,17,18,19,27]. The suppression of AR in SCs and other somatic cells of ETRs in the current study (Figure 1) may be related to androgen suppression [4,14,17,27]. This suppression of AR in SCs of ETRs in stages VII–VIII was associated with enhanced autophagic response based on LC3 expression, TEM and double labeling of LC3 with pan cathepsin (Figure 2, Figure 3, Figure 4 and Figure 5) [4]. Because germ cells do not express AR, the action of androgens on spermatogenesis is dependent on their binding to SCs AR [18]. It has been reported that secretion of essential proteins required for spermatogenesis are controlled by androgens specifically at stages VI–VIII of the spermatogenic cycle [13]. Interestingly, an AR antagonist (bicalutamide) was found to upregulate prosurvival autophagy in LNCaP prostate cancer cells [28]. In consideration of these observations, it can be considered that induction of AR suppression may activate a compensatory prosurvival autophagic response in expressing cells such as SCs, which may be essential for supporting germ cells by required proteins. Further studies are needed for clarification.

As AR signaling from SC nuclei is essential for spermiation and germ cell maturation and survival [18,27], the enhanced germ cell apoptosis and sperm retention observed during stages VII–VIII in ETR testes (Figure 6) may be related to AR suppression, resulting in activation of LAP in autophagic SCs [4,7]. The suppression of androgens and AR may also cause mitochondrial damage in SCs of ETRs during the same stages as recently reported in various tissues [29,30]. However, mitochondrial damage in SCs, as evidenced by PINK1 accumulation in SCs of ETRs (Figure 7), may be accelerated by other mechanisms such as ethanol-induced oxidative stress and iNOS-related nitrative stress [2,3,4,5,6].

The current study showed the accumulation of numerous large LDs in SCs of ETRs, which may be related to enhanced phagocytosis of germ cells and RBs (21], androgen suppression [22,23] and associated mitochondrial damage [24]. The observation of large LDs and damaged mitochondria in the spermiation stage may stimulate bulk autophagy and/or selective autophagy of LDs (lipophagy) [1,2,5] and mitochondria (mitophagy) [3,6]. Further studies are needed to clarify the involvement of PINK1-Parkin related mitophagy and lipophagy in SC responses to various toxicant situations such as acute and chronic alcohol intake. 

Autophagy and mitophagy upregulation in SCs of ETRs during the androgen-dependent stages may support survival by clearing pro-apoptotic damaged mitochondria and excessive toxic LDs [2,3,4,5,6,31], releasing sperm via ES assembly [9], supplying germ cells with anti-apoptotic lactate [4,10,11] and promoting phagocytosis of apoptotic germ cells [7]. This may be essential for the maintenance of testicular homeostasis in the presence of toxic insults such as excessive ethanol intake, thereby preventing infertility associated with enhanced germ cell apoptosis and spermiation failure.

## 4. Materials and Methods

### 4.1. Study Approval

Adult male Wistar rats were treated in keeping with the relevant Experimental Animal Research Committee of Osaka Medical College guidelines (approved by the Animal Research Committee of Osaka Medical College on 28 October 2013, under code 25090).

### 4.2. Antibodies and Kits

The following primary antibodies were used: rabbit anti-LC3B antibody (PM036) from MBL, Nagoya, Japan; rabbit anti-PINK1 (NB600-973) from Novus Biologicals; goat anti-pan-cathepsin (sc-6499), Rabbit anti-AR (sc-816) antibodies, goat anti-Actin (sc-1616) from Santa Cruz (Dallas, TX, USA). For immunofluorescence, the following secondary antibodies were used: Alexa Fluor 594-conjugated secondary antibodies (Molecular Probes, Carlsbad, CA, USA) and VectaFluor™ R.T.U. DyLight^®^ 488 (DI-3788) from Vector, CA, USA. A TUNEL kit (Roche Diagnostics, Mannheim, Germany) was used for apoptosis detection. Vectastain ABC Standard Kit (PK-4000) and ImmPACT DAB(SK-4105) from Vector were used for IHC. Donkey anti-rabbit IgG-HRP, Santa Cruz (sc-2077) was used for Western blot as secondary antibody.

### 4.3. Animal Experiment

The animals were given a single intraperitoneal dose of ethanol (5 g/kg) (this dose of ethanol represents a human binge drinking model) [3,4], and a control group was given the same volume of physiological saline. The animals were sacrificed, and testes were taken at 0, 3, 6, and 24 h after ethanol administration (three animals for each group). The time period 24 h was selected in this study as it showed the highest autophagic activity as previously reported [4]. Testes were fixed in either 4% paraformaldehyde for light microscopy and embedding in paraffin or 2% paraformaldehyde and 2.5% glutaraldehyde in 0.1 M phosphate buffer for TEM [3,4,6]. Unfixed testicular tissues were frozen in liquid nitrogen for western blot analysis.

### 4.4. Immunohistochemical Staining for AR, LC3 and PINK1

The methods for immunohistochemical staining of AR, LC3 and PINK1 were performed according to the manufacturer’s recommendations and our recent study [4]. From paraffin blocks, 4-µm-thick sections were cut and underwent a process of deparaffinization, antigen retrieval, blocking of endogenous peroxidase activity, and incubation for 1 h at room temperature with primary antibodies for AR, LC3, and PINK1. Immunostaining was performed using ABC method. Briefly, Vectastain ABC reagents were applied on sections, followed by biotinylated secondary antibody and DAB. The sections were observed under an Olympus BX41microscope (BX41, Olympus, Tokyo, Japan).

### 4.5. Quantitative Analysis for LC3 Expression

For quantification for LC3 immunostaining in SCs at stages I–VI, VII–VIII, IX–XI, and XII–XIV, 10–15 seminiferous tubules per stage group were used from ETRs and the control group. Using Adobe Photoshop, the tubules were captured and saved for computer analysis using Image J (National Institutes of Health, Bethesda, MA, USA). The LC3 intensity in SCs were identified using the threshold feature of Image menu of the ImageJ program as reported by us [32] and others [33,34]. All quantitative values were expressed as the means ± standard deviation (SD) of the expressions in various stages of spermatogenic cycle. Statistical significance was evaluated using a Student’s *t*-test. *p* values of <0.05 were considered statistically significant.

### 4.6. Immunofluorescence Double-Labeling of LC3/Pan-Cathepsin

For double-labeling of LC3 and pan-cathepsin, we used a sequential method as previously reported [2,3,4]. Following incubation with primary antibodies for 1 h, Alexa Fluor 594 (Molecular Probes, Carlsbad, CA, USA) and VectaFluor™ R.T.U. DyLight^®^ 488 were used as secondary reagents (30 min). The sections were observed under the Olympus BX41 immunofluorescence microscope after nuclear counterstaining with DAPI (blue color) (Vector).

### 4.7. TUNEL Assay for Apoptosis Detection

Terminal deoxynucleotidyl transferase dUTP-mediated nick-end labelling (TUNEL) was performed according to the manufacturer’s protocols, as previously reported [2,3,4]. TUNEL positive cells showed green reaction under immunofluorescence microscope, while TUNEL negative nuclei appeared blue with 4′,6-diamidino-2-phenylindole (DAPI).

### 4.8. TEM

Ultrathin sections (70 nm) from blocks embedded in epoxy were cut, double-stained with uranyl acetate and lead citrate, and examined using an H-7650 transmission electron microscope. The ultrastructural features of apoptosis, autophagy and mitophagy were defined as reported by us and others [2,4,5,6,35,36,37].

### 4.9. Western Blot Analysis

In brief, after homogenization and centrifugation of whole testicular tissues, the supernatant was electrophoresed on 12% sodium dodecyl sulfate polyacrylamide gel and transferred onto a polyvinylidene difluoride membrane. Proteins were detected with the specific primary antibodies for AR and PINK1, and then with specific peroxidase-labelled secondary antibodies as previously reported [4]. The relative intensity of expression of various proteins against actin was normalized and densitometrically measured using Image J.

## 5. Conclusions

Stage-specific enhancement of autophagic and mitophagic responses in SCs in response to ethanol toxicity is marked in stages VII–VIII, which may be related to AR suppression, accumulation of damaged mitochondria and LDs and phagocytosis of germ cells. This may be essential for supplying remaining germ cells with antiapoptotic factors such as lactate and for stimulation of spermiation, thereby maintaining fertility during these critical stages, specifically with exposure to various toxic insults such as alcohol abuse.
